# Characteristics of atheromatosis in the prediabetes stage: a cross-sectional investigation of the ILERVAS project

**DOI:** 10.1186/s12933-019-0962-6

**Published:** 2019-11-15

**Authors:** Enric Sánchez, Àngels Betriu, Carolina López-Cano, Marta Hernández, Elvira Fernández, Francisco Purroy, Marcelino Bermúdez-López, Cristina Farràs-Sallés, Silvia Barril, Reinald Pamplona, Ferran Rius, Cristina Hernández, Rafael Simó, Albert Lecube, the ILERVAS project collaborators, Ferran Barbé, José-Manuel Valdivielso, Glòria Arqué, Jessica González, Ana Vena, Eva Miquel, Marta Ortega-Bravo, Gerard Torres, Serafín Cambray, Manuel Portero-Otin, Mariona Jové, Montserrat Martínez-Alonso, Eva Castro, Pere Godoy

**Affiliations:** 1 Endocrinology and Nutrition Department, University Hospital Arnau de Vilanova, Obesity, Diabetes and Metabolism (ODIM) research Group, IRBLleida, University of Lleida, Lleida, Spain; 2 Vascular and Renal Translational Research Group, IRBLleida, RedinRen-ISCIII, Lleida, Spain; 3 Stroke Unit, University Hospital Arnau de Vilanova, Clinical Neurosciences Group. IRBLleida, University of Lleida, Lleida, Spain; 4 Applied Epidemiology Research Group, IRBLleida, Lleida, Spain; 5 Unitat de Suport a la Recerca Lleida, Fundació Institut Universitari per a la recerca a l’Atenció Primària de Salut Jordi Gol i Gurina (IDIAPJGol), Barcelona, Spain; 6 Respiratory Department, University Hospital Arnau de Vilanova-Santa María, Translational Research in Respiratory Medicine, IRBLleida, University of Lleida, Lleida, Spain; 7 Centro de Investigación Biomédica en Red de Enfermedades Respiratorias (CIBERES), Instituto de Salud Carlos III (ISCIII), Madrid, Spain; 8 Experimental Medicine Department, IRBLleida, University of Lleida, Lleida, Spain; 9 Endocrinology and Nutrition Department, University Hospital Vall d’Hebron. Diabetes and Metabolism Research Unit, Vall d’Hebron Institut de Recerca (VHIR), Autonomous University of Barcelona, Pg. Vall d’Hebron 119-129, 08024 Barcelona, Spain; 10 Centro de Investigación Biomédica en Red de Diabetes y Enfermedades Metabólicas Asociadas (CIBERDEM), Instituto de Salud Carlos III (ISCIII), Madrid, Spain

**Keywords:** Cardiovascular risk factors, Glycosylated hemoglobin, Subclinical atheromatous disease, Prediabetes

## Abstract

**Background:**

Prediabetes has recently been associated with subclinical atheromatous disease in the middle-aged population. Our aim was to characterize atheromatous plaque burden by the number of affected territories and the total plaque area in the prediabetes stage.

**Methods:**

Atheromatous plaque burden (quantity of plaques and total plaque area) was assessed in 12 territories from the carotid and femoral regions using ultrasonography in 6688 non-diabetic middle-aged subjects without cardiovascular disease. Prediabetes was defined by glycosylated hemoglobin (HbA1c) between 5.7 and 6.4% according to the American Diabetes Association guidelines.

**Results:**

Prediabetes was diagnosed in 33.9% (n = 2269) of the ILERVAS participants. Subjects with prediabetes presented a higher prevalence of subclinical atheromatous disease than participants with HbA1c < 5.7% (70.4 vs. 67.5%, p = 0.017). In the population with prediabetes this was observed at the level of the carotid territory (p < 0.001), but not in the femoral arteries. Participants in the prediabetes stage also presented a significantly higher number of affected territories (2 [1;3] vs. 1 [0;3], p = 0.002), with a positive correlation between HbA1c levels and the number of affected territories (r = 0.068, p < 0.001). However, atheromatosis was only significantly (p = 0.016) magnified by prediabetes in those subjects with 3 or more cardiovascular risk factors. The multivariable logistic regression model showed that the well-established cardiovascular risk factors together with HbA1c were independently associated with the presence of atheromatous disease in participants with prediabetes. When males and females were analyzed separately, we found that only men with prediabetes presented both carotid and femoral atherosclerosis, as well as an increase of total plaque area in comparison with non-prediabetic subjects.

**Conclusions:**

The prediabetes stage is accompanied by an increased subclinical atheromatous disease only in the presence of other cardiovascular risk factors. Prediabetes modulates the atherogenic effect of cardiovascular risk factors in terms of distribution and total plaque area in a sex-dependent manner.

*Trial registration* NCT03228459 (clinicaltrials.gov)

## Background

The International Diabetes Federation has estimated that in 2017 there were 451 million individuals with diabetes plus 374 million people with impaired glucose tolerance worldwide, with a total expenditure of USD 850 billion [1]. Cardiovascular (CV) disease is the main comorbidity of diabetes and is estimated to affect 32.2% of all subjects and is responsible for 27% of the total cost of treating diabetes [2, 3]. At diagnosis, many patients with type 2 diabetes have one or more additional classical risk factors for macrovascular disease and many have evidence of overt atherosclerosis [4]. In addition, all phases in the pathophysiology of plaque formation are enhanced in type 2 diabetes, contributing to an accelerated process [5]. However, symptoms are not always present, and the term “unrecognized diabetic cardiac impairment” has been proposed for individuals that develop CV disease without the classic angina-related or heart failure symptoms [6]. Finally, compared with subjects without diabetes, atheromatous disease in diabetes has special characteristics, such as its being more extensive, and affecting multiple and more peripheral blood vessels, that makes it more serious and aggressive [7].

The problem increases when we consider that many of the atherogenic risk factors are already present in the prediabetic stage, years before the diagnosis of type 2 diabetes [8]. The milieu that favors CV disease in the prediabetes stage is more than hyperglycemia in the nondiabetic range and the effect of insulin resistance in the vessel walls. It includes a cluster of different metabolic changes that favors the development of atheromatous disease, such as low-grade chronic inflammation, endothelial vasodilator and fibrinolytic dysfunction, and an atherogenic lipoprotein profile [9, 10]. In this way, prospective cohort studies have shown how prediabetes [defined as impaired fasting glucose, impaired glucose tolerance or raised glycated hemoglobin (HbA1c)] is associated with an increased risk of composite CV disease, coronary heart disease and stroke compared with normoglycemia [11]. Similarly, during an 8-years period and compared to individual with persistent normoglycemia, those who shifted from normoglycemia to impaired fasting glucose had a significant increased risk of all-cause mortality [12]. Remarkably, the health risk increases in subjects with values as low as 5.6 mmol/L for fasting glucose concentration or 39 mmol/mol for HbA1c. On this basis, it is of clinical relevance to evaluate the characteristics of subclinical atheromatous disease in the prediabetes stage [11].

To shed light on this issue, we performed a cross-sectional study in order to characterize atheromatous plaque burden by the number of affected territories and the total plaque area in the prediabetes stage.

## Methods

### Study population, metabolic status and the selection of patients

A total of 6809 subjects were enrolled between January 2015 and December 2017 from 30 primary health care centers in Lleida, Spain. The ILERVAS project is an ongoing clinical trial dealing with subclinical atheromatous disease (ClinicalTrials.gov Identifier: NCT03228459) [13]. The inclusion criteria were as follows: age 45–70 years, no history of cardiovascular disease, and at least one cardiovascular risk factor (dyslipidemia, blood hypertension, obesity, smoking habit or a first degree relative with premature (< 55 years old in men, < 65 in women) cardiovascular disease (myocardial infarction, stroke and peripheral arterial disease). The exclusion criteria were any type of diabetes, chronic kidney disease, active neoplasia, a life expectancy of less than 18 months and pregnancy.

According to the current American Diabetes Association guidelines, prediabetes was defined as an HbA1c between 39 and 47 mmol/mol (5.7 to 6.4%) and normal glucose metabolism as an HbA1c < 39 mmol/mol (< 5.7%) [14]. The HbA1c test was performed in capillary blood using a point-of-care device [Cobas B 101^®^, Roche Diagnostics S.L., Sant Cugat del Vallès, Spain], based on a latex agglutination inhibition immunoassay procedure that meets the generally accepted performance criteria for HbA1c [15]. A total of 121 participants with previously undiagnosed type 2 diabetes [(HbA1c ≥ 48.0 mmol/mol (≥ 6.5%)] were excluded from the investigation that was finally performed in 6688 subjects.

Clinical data regarding the cardiovascular risk factors were obtained from an electronic database (*Information System for the Development of Research in Primary Care*, *SIDIAP*) that comprises anonymized and longitudinal information from the Catalan Health Institute. The prevalence of dyslipidemia was obtained from patients who during the study period had a diagnostic code for disorders of lipoprotein metabolism according to of the International Classification of Diseases codes. The incidence of blood hypertension was obtained from subjects who had an identification code for hypertensive diseases. Obesity was defined by a body mass index (BMI) ≥ 30 kg/m^2^.

The prescribed antihypertensive, lipid-lowering and antithrombotic treatments were extracted from prescription- and pharmacy-invoicing databases provided by the *Catalan Health Service*, which are incorporated yearly into the SIDIAP database. Antihypertensive medication agents included ACE inhibitors, diuretics, ARA II, beta-blockers, calcium antagonists and other antihypertensives. Lipid-lowering drugs included statins, fibrates, ezetimibe and omega-3 fatty acids. Antithrombotic treatment consisted of the use of anticoagulant or antiplatelet agents.

### Evaluation of clinical variables

Height and body weight were measured without shoes and in light clothing, and the body mass index (BMI) was obtained. A non-stretchable tape with a precision of 0.1 cm was used to assess waist circumference. According to a specified protocol, total cholesterol (mg/dl) levels were assessed in all participants from a non-fasting dried capillary blood test (fingertip puncture) using the REFLOTRON^®^ Plus system (Roche Diagnostics, GmbH, Germany) [13]. Quantification of the entire lipid profile (HDL cholesterol, LDL cholesterol and triglycerides) was evaluated only in subjects in which total cholesterol was ≥ 200 mg/dL after fasting for 6 h or where total cholesterol ≥ 250 mg/dL regardless of fasting hours.

Blood pressure was measured in triplicate, after 5 min’ rest using an automated device [Omron M6 Comfort HEM-7221-E (Omron Healthcare, Kyoto, Japan)] at 2-min intervals, and the mean of the last 2 was calculated. The smoking habit (non-smoker, current or former smoker) was also documented. Smokers who stopped smoking ≥ 1 year prior to recruitment were considered former smokers.

### Assessment of atheromatous plaque burden by ultrasound study

Bilateral carotid (common, bifurcation, internal and external arteries) and femoral (common and superficial arteries) areas were explored. The pictures were obtained by qualified sonographers using an ultrasonic Doppler Ultrasound Vivid-I (General Electric Healthcare, Waukesha, WI, USA) equipped with probe broadband linear 12L-RS that works at frequencies between 5 and 13 MHz. Standardized and validated scanning and reading protocols were used to decrease inter-operator variability and type 2 errors [16]. To measure intra and inter rater absolute agreement, Fleiss’ kappa for plaque presence and intraclass correlation coefficient for plaque area were obtained (Additional file 1: Table S1). The readers were unaware of the patients’ clinical histories.

Subclinical atheromatosis was defined as the presence of any plaque in the twelve assessed areas [17]. A plaque was well-defined as a focal intima-media thickness ≥ 1.5 mm protuberant in the lumen [18]. Subjects were categorized as having focal (1 territory), intermediate (2 to 3 territories), or generalized (4 to 12 territories) atheromatous disease. All plaques were measured, and the total plaque area (cm^2^) was assessed [19].

### Statistical analysis

Owing the non-normal distribution of the data detected by the Shapiro–Wilk test, quantitative data was expressed as the median [interquartile range]. Comparisons between the prediabetes and non-prediabetes groups were made using the Mann–Whitney U test for quantitative variables, and the Pearson’s Chi-squared test for categorical variables. The relationship between continuous variables was assessed by the Spearman correlation test.

A multivariable logistic regression model for the presence of subclinical atheromatous disease for the development cohort was performed including the following confounding elements: sex, age, HbA1c, total cholesterol, systolic blood pressure, the BMI, waist circumference, smoking habit and medical treatments. The calibration and discrimination of the multivariable logistic regression model were evaluated using the goodness of fit Hosmer–Lemeshow test and the area under the Receiver Operating Characteristic curve, correspondingly. All “p” values were based on a two-sided test of statistical significance, and significance was accepted at the level of p < 0.050. All statistical analyses were performed using SSPS statistical package (IBM SPSS Statistics for Windows, Version 25.0. Armonk, NY, USA).

## Results

From the baseline sample of 6809 subjects, prediabetes was diagnosed in 33.3% (n = 2269) of the subjects. The main clinical and metabolic data according to the HbA1c values are displayed in Table 1. Participants with prediabetes were older and presented a higher ratio of women and classical CV risk factors such as dyslipidemia, hypertension and obesity in comparison with the control group. The prevalence of subclinical atheromatous disease in the entire population was significantly higher in subjects with prediabetes than in control participants (70.4% vs. 67.5%, p = 0.017). This difference was at the expense of carotid territory (49.3% vs. 43.5%, p < 0.001), and disappeared when only the femoral territory was evaluated (54.1% vs. 52.5%, p = 0.228). When each of the six specific territories was analyzed in the left and right side, slightly marked differences were observed in the left arteries (Table 2). In addition, when only women were analyzed, the increased prevalence of subclinical atheromatous disease detected in postmenopausal control women when compared to premenopausal control women appeared to be attenuated among those with prediabetes (Additional file 1: Table S2).

**Table 1:** Main clinical, metabolic data, medical treatment and atheromatous disease characteristics in the study population according to the presence of prediabetes

	Prediabetes (n = 2269)	Control group (n = 4419)	p
Women, n (%)	1290 (56.9)	2151 (48.7)	< 0.001
Age (years)	59 [54;64]	57 [52;62]	< 0.001
HbA1c (mmol/mol)	40 [39;42]	36 [33;37]	< 0.001
HbA1c (%)	5.8 [5.7;6.0]	5.4 [5.2;5.5]	< 0.001
Dyslipidemia, n (%)	1290 (56.9)	2214 (50.1)	< 0.001
Total cholesterol (mg/dL)	206 [183;231]	203 [179;229]	0.002
LDL-cholesterol^a^ (mg/dL)	146 [133;164]	145 [129;162]	0.020
HDL-cholesterol^a^ (mg/dL)	53 [45;65]	55 [46;67]	0.009
Triglycerides^a^ (mg/dL)	148 [113;205]	133 [100;183]	< 0.001
Lipid-lowering agents, n (%)	534 (23.5)	712 (16.1)	< 0.001
Statins, n (%)	478 (21.0)	656 (14,8)	< 0.001
Fibrates, n (%)	62 (2.7)	54 (1.2)	< 0.001
Ezetimibe, n (%)	8 (0.3)	17 (0.3)	0.838
Omega-3 fatty acids, n (%)	6 (0.2)	0 (0.0)	0.001
Hypertension, n (%)	1048 (46.2)	1617 (36.6)	< 0.001
Systolic BP (mm Hg)	132 [120;142]	129 [119;141]	< 0.001
Diastolic BP (mm Hg)	81 [75;88]	81 [75;88]	0.764
Pulse pressure (mm Hg)	49 [42;58]	47 [40;56]	< 0.001
Antihypertensives, n (%)	911 (40.1)	1305 (29.5)	< 0.001
ACE inhibitors, n (%)	430 (18.9)	659 (14.9)	< 0.001
ARA II, n (%)	230 (10.1)	334 (7.5)	< 0.001
Diuretics, n (%)	441 (19.4)	586 (13.2)	< 0.001
Beta-blockers, n (%)	196 (8.6)	216 (4.8)	< 0.001
Calcium antagonists, n (%)	147 (6.4)	210 (4.7)	0.003
Other, n (%)	12 (0.5)	19 (0.4)	0.573
Obesity^b^, n (%)	817 (36.0)	1176 (26.6)	< 0.001
BMI (kg/m^2^)	29.6 [26.7;33.1]	28.0 [25.1;31.2]	< 0.001
Current or former smoker, n (%)	1211 (53.4)	2714 (61.4)	< 0.001
Antithrombotics, n (%)	94 (4.1)	115 (2.6)	0.001
Number of CV risk factors	2 [1;3]	2 [1;2]	< 0.001
Characteristics of atheromatous disease			
Presence of any plaque, n (%)	1597 (70.4)	2984 (67.5)	0.017
Carotid territory affected, n (%)	1112 (49.0)	1924 (43.5)	< 0.001
Femoral territory affected, n (%)	1227 (54.1)	2321 (52.5)	0.228
Number of affected territories	2 [1;3]	1 [0;3]	0.002
Total plaque area, (cm^2^)	0.96 [0.52;1.62]	0.93 [0.56;1.55]	0.760
Stenotic lesions ≥ 50%	21 (0.9)	23 (0.5)	0.057

Data are expressed as a median [interquartile range] or n (percentage)

*HbA1c* glycosylated hemoglobin, *LDL* low density lipoprotein, *HDL* high density lipoprotein, *BP* blood pressure, *BMI* body mass index, *CV* cardiovascular

^a^Determination was done in cases in which total cholesterol was ≥ 200 mg/dL and after fasting for 6 h or total cholesterol ≥ 250 mg/dL regardless of fasting hours

^b^Obesity was defined as a BMI ≥ 30 kg/m^2^

**Table 2:** Prevalence of subclinical atheromatous disease in each of the six specific territories in left and right arteries according to the presence of prediabetes

	Prediabetes (n = 2269)	Control group (n = 4419)	p
Left arteries	1297 (57.2)	2395 (54.2)	0.021
Common carotid artery, n (%)	95 (4.2)	142 (3.2)	0.041
Bifurcation carotid artery, n (%)	644 (28.4)	1118 (25.3)	0.018
Internal carotid artery, n (%)	350 (15.4)	546 (12.3)	0.002
External carotid artery, n (%)	31 (1.4)	69 (1.6)	0.534
Common femoral artery, n (%)	925 (40.8)	1775 (40.2)	0.636
Superficial femoral artery, n (%)	152 (6.7)	232 (5.3)	0.016
Right arteries	1348 (59.4)	2513 (56.9)	0.046
Common carotid artery, n (%)	68 (3.0)	129 (2.9)	0.376
Bifurcation carotid artery, n (%)	659 (29.1)	1053 (23.8)	< 0.001
Internal carotid artery, n (%)	322 (14.2)	578 (13.1)	0.240
External carotid artery, n (%)	40 (1.8)	78 (1.8)	0.992
Common femoral artery, n (%)	1018 (44.9)	1936 (43.8)	0.411
Superficial femoral artery, n (%)	172 (7.6)	275 (6.2)	0.035

Data are expressed as a median [interquartile range] or n (percentage)

We observed a significant correlation between HbA1c levels and the number of affected territories (r = 0.068, p = 0.001), but not with the total plaque area (r = − 0.008, p = 0.609). Participants with prediabetes almost increased twice the number of affected territories with plaque [2 (1 to 3) vs. 1 (0 to 3) territories, p = 0.002) compared with control subjects and, therefore, were more likely to be classified as individuals with generalized atheromatous disease (19.5% vs. 16.3%, p < 0.001). However, the vast majority (82.6%) presented less than 4 affected territories. In addition, no differences in the carotid plaque area (0.22 [0.12;0.42] vs. 0.20 [0.11;0.41], p = 0.555), the femoral plaque area (0.68 [0.29;1.24] vs. 0.68 [0.34;1.18], p = 0.683) or the total plaque area (0.96 [0.52;1.62] vs. 0.93 [0.56;1.55], p = 0.760) were observed between groups.

**Fig. 1:**
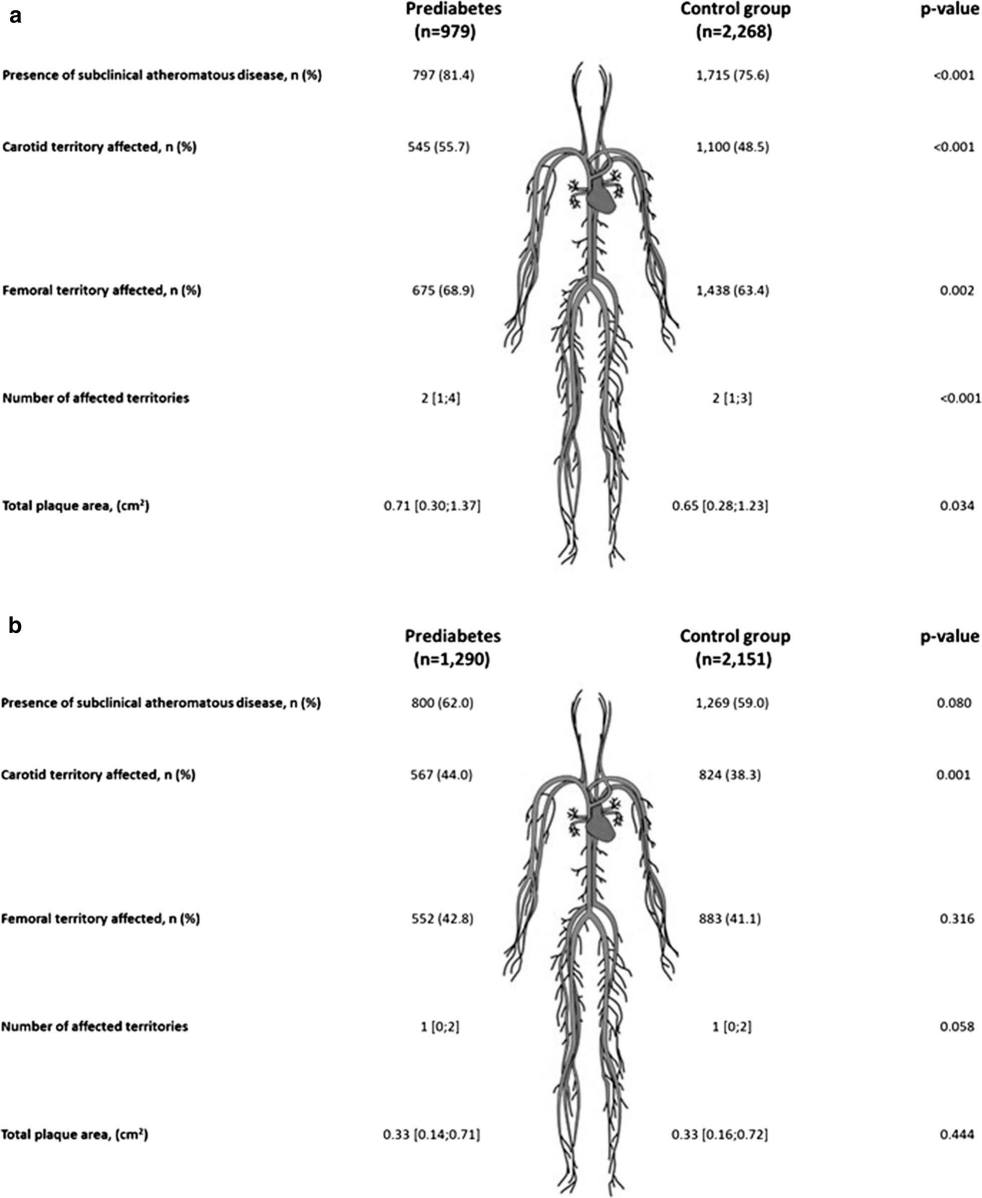
Atheromatous disease characteristics in the; **a** men and **b** women. Data are expressed as a median [interquartile range] or n (percentage)

When the results were assessed according to sex, the differences regarding subclinical atheromatous disease were greater among males than females (Fig. 1). Men with prediabetes presented not only a higher number of plaques in both the carotid and femoral regions but also an increase of affected territories and total plaque area in comparison with age-matched individuals without prediabetes. By contrast, women with prediabetes only presented a higher number of plaques in the carotid region and a similar number of affected territories and total plaque area to age-matched women without prediabetes.

**Fig. 2:**
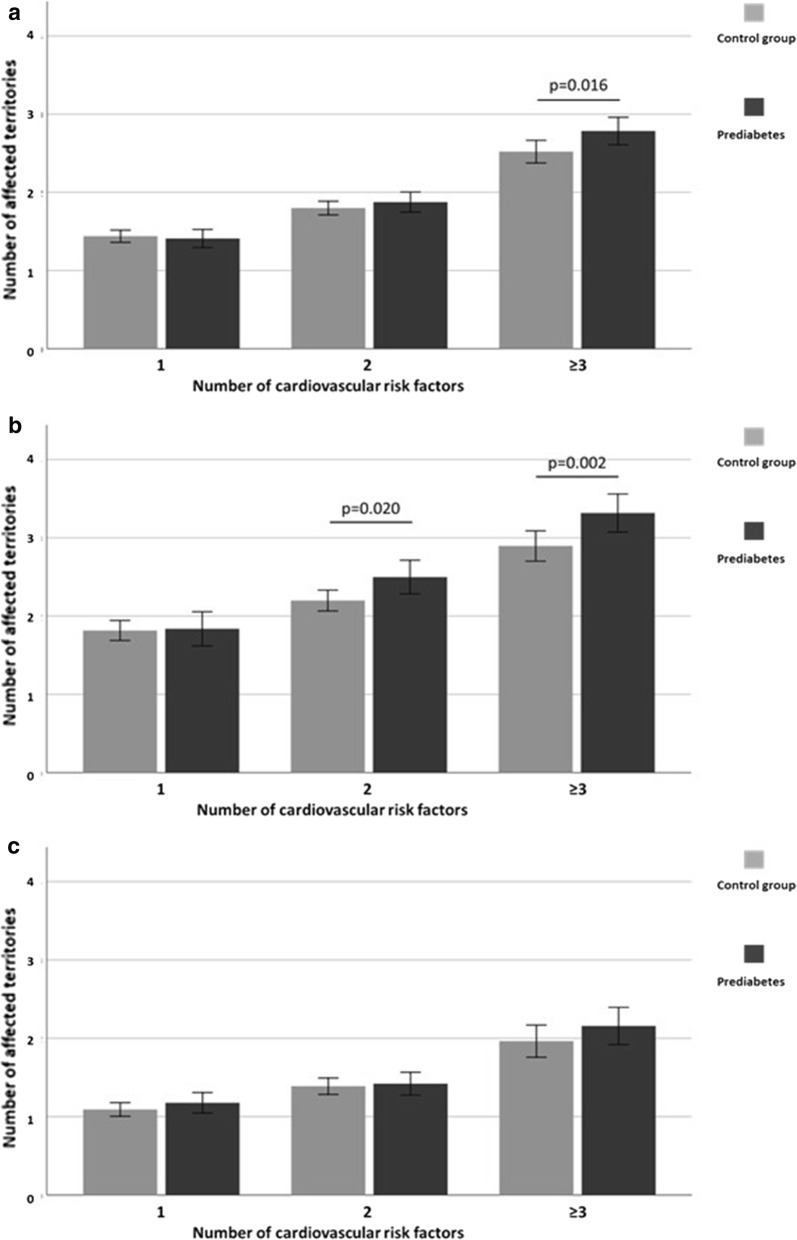
Plot displaying the number of affected territories with atheromatous plaque according to the quantity of cardiovascular risk factors such as dyslipidemia, blood hypertension, obesity, smoking habit or a first degree relative with premature cardiovascular disease in the; **a** entire population, **b** men and **c** women

In the entire population, the quantity of cardiovascular risk factors was correlated with the number of affected territories with atheromatous plaque (r = 0.223, p < 0.001) and with the total plaque area (r = 0.192, p < 0.001). However, the presence of prediabetes only impacted on the burden of subclinical atheromatous disease when subjects presented three or more CV risk factors **(**Fig. 2a**)**. Notably, when the analysis was performed taking sex into account, we observed that men with prediabetes and two or more CV risk factors displayed a significantly higher number of affected territories with atheromatous plaque in comparison with men free of prediabetes (Fig. 2b). By contrast, we did not find any significant differences between women with prediabetes and the control group regardless of the number of CV risk factors (Fig. 2c).

Finally, the multivariable logistic regression model showed that smoking habit, male sex, HbA1c, age, systolic blood pressure, total cholesterol, BMI and lipid-lowering and antihypertensive drugs were independently associated with the presence of atheromatous disease in participants with prediabetes (Table 3). When both sexes were assessed separately, the same parameters excluding HbA1c accounted for subclinical atherosclerosis disease in females with prediabetes (Additional file 1: Table S3), whereas only smoking status, age and systolic blood pressure were the independent variables in males (Additional file 1: Table S4).

**Table 3:** The multivariable logistic regression model for presence of atheromatous disease in subjects in the prediabetes stage

	Odds ratio (95% confidence interval)	p
Sex		
Women	Ref.	
Men	3.37 (2.60 to 4.36)	< 0.001
Age (years)	1.10 (1.07 to 1.12)	< 0.001
Glycosylated hemoglobin (%)	1.92 (1.09 to 3.38)	0.024
Total cholesterol (mg/dL)	1.01 (1.01 to 1.01)	< 0.001
Lipid-lowering agents		
No	Ref.	
Yes	0.64 (0.50 to 0.83)	0.001
Systolic blood pressure (mm Hg)	1.01 (1.01 to 1.02)	< 0.001
Antihypertensive treatment		
No	Ref.	
Yes	0.77 (0.62 to 0.96)	0.018
Body mass index (kg/m^2^)	0.99 (0.96 to 1.01)	0.234
Waist circumference^a^		
Normal	Ref.	
High	1.05 (0.77 to 1.42)	0.762
Smoking status		
Never	Ref.	
Former	1.89 (1.49 to 2.41)	< 0.001
Current	5.23 (3.85 to 7.11)	< 0.001
Antithrombotic treatment		
No	Ref.	
Yes	0.82 (0.46 to 1.45)	0.491
Test of fit Hosmer–Lemeshow		0.586
Area under de ROC curve	0.74 (0.72 to 0.76)	< 0.001

^a^High waist circumference was defined as ≥ 102 cm for men and ≥ 88 cm for women

## Discussion

In the present study we provide evidence that prediabetes modulates the atherogenic effect of cardiovascular risk factors in terms of the distribution of plaques and the total atherosclerotic burden in a sex-dependent manner. In this regard, our results suggest that prediabetes and underlying insulin resistance act as an enhancer of the atherosclerotic process, but only in men with 2 or more classical CV risk factors. In addition, the distribution of plaques occurred mainly in the carotid territory in women whereas a more severe and widespread plaque burden was observed in men.

### Cardiovascular disease in prediabetes

The relationship between prediabetes and subclinical CV disease has been well-documented [20, 21]. In the *Heinz Nixdorf Recall Study*, a population-based cohort of 2184 without overt CV disease from Germany, participants with prediabetes (fasting plasma glucose ≥ 6.1 but < 7.0 mmol/l) showed a higher prevalence of coronary artery calcification than normoglycemic participants [20]. However, the association between impaired fasting glucose and coronary artery calcification was less pronounced in women [20]. In the *Multi*-*Ethnic Study of Atherosclerosis*, a cross-sectional study among 5121 participants without type 2 diabetes or CV disease, those in the highest quartile of HbA1c showed significantly higher values for common and internal carotid intimal-medial wall thickness in both sexes, but the association between HbA1c and carotid artery calcification was only present in women [21]. On the other hand, in a recent study with 6434 asymptomatic Korean individuals who underwent a coronary computed tomographic angiography, prediabetes was not associated with an increased risk of subclinical coronary atherosclerosis [22]. Our study adds information by providing the characteristics of the atheromatous process in a population of middle-aged subjects without previous vascular disease according to the presence of prediabetes. We describe how subjects with prediabetes suffer a higher prevalence of atheromatous disease, mainly in the carotid territories, when compared with subjects with normal HbA1c. In addition, vascular disease in participants with prediabetes is characterized by the presence of plaques of a similar area in a higher number of affected territories. Overall, our data reinforce the idea that CV disease is a progressive defect associated with glucose abnormalities, appearing and increasing throughout the prediabetes stage. In fact, in the subjects with prediabetes, HbA1c positively correlates with the number of plaques. However, it should be noted that in the population included in our study the total plaque area was similar to age-matched control subjects. In addition, prediabetes was only a trigger for the number of affected territories when at least 3 classic CV risk factors were present. These findings seriously call into question the importance of insulin resistance and the prediabetes stage as primary factors of atherogenesis in the type 2 diabetes population. The lack of significance of the waist circumference in the multivariable logistic regression model in both sexes also argues against this assumption. In the *Coronary Artery Risk Development in Young Adults* Study, for each 5-year-long duration of prediabetes the hazard ratio for the presence of coronary artery calcified plaque was only 1.07 (1.01 to 1.13) [23]. This result also supports the modest effect of prediabetes itself as a CV risk factor.

The different impact of prediabetes on atherosclerotic processes in men and women merits some comment. First, it has been suggested that men and women may progress from normoglycemia to overt type 2 diabetes by different ways: more men than women had impaired fasting glucose, whereas women more often had impaired glucose tolerance [24]. Also, in the *Framingham Heart Study*, the 4-year coronary heart disease event rate among participants with prediabetes differed according to the prediabetes definition and the participants’ sex [25]. Whether or not the different transitional stage from normoglycemia to diabetes has a differential effect in the atherogenic process is an issue that remains to be elucidated. Second, the relative contribution of ageing and menopause itself in the development of CV diseases still remains uncertain [26]. It should be noted that the youngest women recruited in the LERVAS cohort were 50 years old. Therefore, the relative protection of women against prediabetes-induced plaque formation seems to be unrelated with estrogen levels. However, the inherent difference in circulating testosterone levels between men and women could not be ruled out as an underlying mechanism accounting for the widespread and significantly greater plaque detected in men [27].

The predilection of prediabetes for the carotid territory deserves attention, particularly when its impact in CV disease may be markedly different. In an autopsy study, Dalager et al. described different features in the microscopic sections of the carotid and superficial femoral arteries, probably reflecting different formation pathways [28]. Unlike the femoral arteries, the carotid bifurcation was prone to foam cell lesions and plaque formation, and lipid core plaques were more much common in the carotid territory in samples from patients who died of coronary atherosclerosis [28]. Similarly, in vivo non-invasive magnetic resonance plaque-imaging visualized significant differences in plaque composition, with larger necrotic cores as well as hemorrhaged areas in the carotid arteries compared to the femoral arteries [29]. And in clinical practice, the significant correlation found between the stenosis of coronary segments and carotid plaque occurrence disappeared when femoral plaques were evaluated [30]. Therefore, carotid territories are more prone to develop complicated plaques than femoral arteries, thus explaining different rates in the progression of atherosclerotic disease and the outcomes of CV events in subjects with prediabetes [11, 31]. In this way, results from the *Emerging Risk Factors Collaboration* revealed that the risk of stroke in patients with diabetes mellitus is increased twofold compared with individuals without diabetes mellitus [32]; the risk of recurrent stroke is also increased [33]. Prediabetes (defined as impaired glucose tolerance or a combination of impaired fasting glucose plus impaired glucose tolerance) has also been associated with a higher future risk of stroke (relative risk 1.20, 95% confidence interval 1.07 to 1.35) [34]. The 10-year planned follow-up of the ILERVAS Project will give us relevant clinical information on this topic [13].

Prediabetes duration has been associated with subclinical atherosclerosis, suggesting that prevention strategies to reverse cumulative exposure to this metabolic transition stage are needed [35]. From the global 374 million people with prediabetes in 2017, only 1.95% will progress to type 2 diabetes per year, suggesting that a substantial number of subjects will continue unprotected against long-term intermediate glycemic elevations [1, 36]. Our results suggest that men with at least 2 traditional risk factors represent the target prediabetes sub-population in which the efforts for testing the protective effects of anti-atherogenic therapeutic strategies should be addressed.

## Potential pathogenic mechanisms

The mechanisms which explain the burden of arterial plaque associated with prediabetes, and their impact on different arterial territories, are not yet fully understood. The role of hemodynamic features and the underlying blood vessel structure warrants attention, since vascular anatomy is not uniform for the thickness of intima-media layer and the left carotid artery originates directly from the aortic arch and is therefore exposed to a constantly higher shear stress [37, 38]. In contrast to the femoral artery, a transitional zone has been described in the carotid bifurcation, an artery segment between elastic and muscular cell types where foam cell lesions and lipid core plaque develop at early ages [28, 39]. In addition, in vivo magnetic resonance imaging studies have described how the decrease of the lumen area by femoral plaque progression is compensated for by positive remodeling, creating a dissimilar rate of progression of luminal stenosis between the carotid and femoral arteries [40]. The impact of metabolic changes associated with prediabetes in these selected segments, such as insulin resistance, low-grade chronic inflammation, advanced glycation end-products (AGEs) production, dyslipidemia or fibrinolytic dysfunction, in the carotid transitional zone or in arterial remodeling is unclear [41–44]. The concentration of the endogenous secretory receptor for the AGEs receptor in 220 patients with prediabetes was significantly lower than in 99 control subjects and was one of the main determinants of the intima-media thickness of the common carotid artery [42]. Similarly, insulin resistance indices were strongly related to carotid intima-media thickness, and plaque presence and area in patients without diabetes, but were without relevance when femoral atherosclerosis was evaluated [41]. More recently, Altin et al. [43] showed how common carotid, but not femoral, intima media thickness was significantly higher in 113 patients with insulin resistance (homeostasis model assessment index > 2.5) free of CV disease compared to 112 controls. In our population without previous CV events, male participants with prediabetes exhibited a higher prevalence of atheromatous plaque both in carotid and femoral territories in comparison with the control group. However, women with prediabetes only presented a higher number of plaques in the carotid region. These findings reinforce the rationale of selecting the carotid territory for performing a screening of subclinical CV disease in those subjects with prediabetes.

### Limitations and strengths

This study has some limitations that need to be considered. First, it is a cross-sectional analysis, so the nature of the study has not allowed us to establish causality. However, we will be doing a follow-up of the entire population until 2028. Second, the three accepted prediabetes definitions according to the criteria of the American Diabetes Association seem to identify different populations, as often not all the tests identify prediabetes in the same individual [14, 45, 46]. Some advantages have been linked to HbA1c, such as that fasting is not required, and the nonappearance of daily alterations in periods of illness or stress, and its higher preanalytical stability [20]. In addition, the prediabetes HbA1c-based definitions appears to be more specific and to provide modest improvements in risk discrimination for CV disease and other clinical complications than definitions based on fasting plasma glucose [47]. Third, although we have tested HbA1c using a point-of-care instrument, the large population included in our study allowed us to define two well differentiated populations, not only in their HbA1c values but also in their anthropometric and clinical characteristics. In addition, we have no available data regarding the duration of prediabetes in our population. Finally, LDL-cholesterol was assessed regardless of the fasting state when total cholesterol was ≥ 200 mg/dl or only after 6 h of fasting when ≥ 250 mg/dl, what it is not the standard recommendation. However, it is not very far from the recent new lipid guidelines that summarizes that in adults ≥ 20 years old and not on lipid-lowering therapy (the 80.7% of our population), measurement of either fasting or a non-fasting lipid profile is effective in estimating atherosclerotic cardiovascular disease risk and recording baseline LDL-cholesterol [48].

## Conclusions

In summary, prediabetes is associated with a significantly increased burden of atheromatous disease in men with 2 or more CV risk factors. This finding points to this subpopulation as the main target for strategies aimed at reducing CV risk factors and HbA1c. In addition, our results suggest that the global impact of prediabetes on the atherosclerotic process in the entire population is quite limited.

## Supplementary information

**Additional file 1: Table S1.** Full results and methodological details regarding intra and inter rate reliability for plaque presence and plaque area by arterial territory. **Table S2.** Prevalence of subclinical atheromatous disease in each of the six specific territories in women according the menopausal state. **Table S3.** The multivariable logistic regression model for the presence of atheromatous disease among female subjects in the prediabetes stage. **Table S4.** The multivariable logistic regression model for the presence of atheromatous disease among male subjects in the prediabetes stage.

## Data Availability

The datasets generated and/or analysed during the current study are not publicly available due to is an ongoing study but are available from the corresponding author on reasonable request.

## Abbreviations

AGEs: advanced glycation end-products
BMI: body mass index
CV: cardiovascular
HbA1c: glycosylated hemoglobin
SIDIAP: Information System for the Development of Research in Primary Care
