# Supplementary material for: Characteristics of atheromatosis in the prediabetes stage: a cross-sectional investigation of the ILERVAS project
Source: Cardiovasc Diabetol. 2019 Nov 15;18:154. doi: 10.1186/s12933-019-0962-6 (PMC6857207; doi:10.1186/s12933-019-0962-6)
Supplement: Supplementary file 1 — Additional file 1: Table S1. Full results and methodological details regarding intra and inter rate reliability for plaque presence and plaque area by arterial territory. Table S2. Prevalence of subclinical atheromatous disease in each of the six specific territories in women according the menopausal state. Table S3. The multivariable logistic regression model for the presence of atheromatous disease among female subjects in the prediabetes stage. Table S4. The multivariable logistic regression model for the presence of atheromatous disease among male subjects in the prediabetes stage. [file 12933_2019_962_MOESM1_ESM.docx]

**Table S1.** Full results and methodological details regarding intra and inter rate reliability for plaque presence and plaque area by arterial territory.

| **Plaque presence** | **Intrarater #1** | | **Intrarater #2** | | **Interrater** | |  |
| --- | --- | --- | --- | --- | --- | --- | --- |
|  | **Fleiss’ kappa (95% CI)** | **N; r** | **Fleiss’ kappa (95% CI)** | **N; r** | **Fleiss’ kappa (95% CI)** | **N** | |
| Right common carotid artery | 1 (1, 1) | 84; 2 | 1 (1, 1) | 84; 2 | 0.73 (0.36, 1) | 80 | |
| Right carotid bulb | 1 (1, 1) | 84; 2 | 1 (1, 1) | 84; 2 | 0.74 (0.58, 0.91) | 80 | |
| Right internal carotid artery | 1 (1, 1) | 84; 2 | 1 (1, 1) | 84; 2 | 0.57 (0.29, 0.86) | 80 | |
| Right external carotid artery | 1 (1, 1) | 84; 2 | 1 (1, 1) | 84; 2 | 1 (1, 1) | 80 | |
| Left common carotid artery | 1 (1, 1) | 84; 2 | 1 (1, 1) | 84; 2 | 0.48 (0, 1) | 80 | |
| Left carotid bulb | 1 (1, 1) | 84; 2 | 1 (1, 1) | 84; 2 | 0.67 (0.46, 0.87) | 80 | |
| Left internal carotid artery | 1 (1, 1) | 84; 2 | 1 (1, 1) | 84; 2 | 0.96 (0.89, 1) | 80 | |
| Left external carotid artery | 1 (1, 1) | 84; 2 | 1 (1, 1) | 84; 2 | 1 (1, 1) | 80 | |
| Right common femoral artery | 1 (1, 1) | 84; 2 | 1 (1, 1) | 84; 2 | 1 (1, 1) | 80 | |
| Right superficial femoral artery | 1 (1, 1) | 84; 2 | 1 (1, 1) | 84; 2 | 0.82 (0.56, 1) | 80 | |
| Left common femoral artery | 1 (1, 1) | 84; 2 | 1 (1, 1) | 84; 2 | 0.97 (0.91, 1) | 80 | |
| Left superficial femoral artery | 1 (1, 1) | 84; 2 | 1 (1, 1) | 84; 2 | 1 (1, 1) | 80 | |
|  | **Intrarater #1** |  | **Intrarater #2** |  | **Interrater** |  | |
| **Plaque area** | **ICC (95% CI)** | **N; r** | **ICC (95% CI)** | **N; r** | **ICC (95% CI)** | **N** | |
| Right common carotid artery | ne | 4; 2 | ne | 4; 2 | ne | 3 | |
| Right carotid bulb | 0.99 (0.97, 0.99) | 19; 2 | 0.96 (0.90, 0.98) | 26; 2 | 0.83 (0.53, 0.94) | 18 | |
| Right internal carotid artery | 0.99 (0.95, 0.99) | 7; 2 | 0.94 (0.76, 0.99) | 15; 2 | 0.80 (0.04, 0.97) | 7 | |
| Right external carotid artery | ne | 4; 2 | ne | 4; 2 | ne | 4 | |
| Left common carotid artery | ne | 1; 2 | ne | 3; 2 | ne | 1 | |
| Left carotid bulb | 0.99 (0.99, 0.99) | 14;2 | 0.98 (0.96, 0.99) | 23; 2 | 0.95 (0.69, 0.99) | 13 | |
| Left internal carotid artery | 1 (1, 1) | 18; 2 | 0.98 (0.93, 0.99) | 17; 2 | 0.97 (0.90, 0.99) | 17 | |
| Left external carotid artery | ne | 1; 2 | ne | 1; 2 | ne | 1 | |
| Right common femoral artery | 1 (0.99, 1) | 32; 2 | 0.99 (0.98, 0.99) | 32; 2 | 0.84 (0.63; 0.94) | 30 | |
| Right superficial femoral artery | ne | 6; 2 | ne | 6; 2 | ne | 5 | |
| Left common femoral artery | 0.99 (0.99, 0.99) | 26; 2 | 0.99 (0.98, 0.99) | 25; 2 | 0.97 (0.91, 0.99) | 25 | |
| Left superficial femoral artery | 1 (1, 1) | 7; 2 | 0.99 (0.48, 1) | 7; 2 | 0.99 (0.95, 1) | 7 | |

ICC estimates and their 95% CI were calculated based on single-measurement, absolute-agreement, 2-way mixed-effects models. ICC: intraclass correlation coefficient; CI: confidence interval; ne: not estimable.

**Table S2.** Prevalence of subclinical atheromatous disease in each of the six specific territories in women according the menopausal state.

|  | **Postmenopausal women** | **Premenopausal women** | **p** |
| --- | --- | --- | --- |
| **Control women group** |  |  |  |
| **n** | 2,078 | 73 | - |
| **Presence of any plaque, n (%)** | 1,243 (59.8) | 26 (35.6) | <0.001 |
| **Carotid territory affected, n (%)** | 809 (38.9) | 15 (20.5) | 0.001 |
| **Femoral territory affected, n (%)** | 863 (41.5) | 20 (27.4) | 0.016 |
| **Number of affected territories** | 2 [1;3] | 2 [1;3] | <0.001 |
| **Total plaque area, (cm^2^)** | 0.33 [0.16;0.72] | 0.36 [0.15;0.70] | 0.941 |
| **Prediabetes women group** |  |  |  |
| **n** | 1,259 | 31 | - |
| **Presence of any plaque, n (%)** | 784 (62.3) | 16 (51.6) | 0.227 |
| **Carotid territory affected, n (%)** | 561 (44.6) | 6 (19.4) | 0.005 |
| **Femoral territory affected, n (%)** | 540 (42.9) | 12 (38.7) | 0.642 |
| **Number of affected territories** | 2 [1;3] | 2 [1;2] | 0.094 |
| **Total plaque area, (cm^2^)** | 0.33 [0.14;0.72] | 0.30 [0.12;0.49] | 0.398 |

Menopause was defined by an arbitrary cut-off age of 51 years old. Data are expressed as a median [interquartile range] or n (percentage).

**Table S3.** The multivariable logistic regression model for the presence of atheromatous disease among female subjects in the prediabetes stage.

|  | Odds Ratio  (95% Confidence Interval) | p |
| --- | --- | --- |
| Age (years) | 1.09 (1.06 to 1.12) | <0.001 |
|  |  |  |
| Glycosylated hemoglobin (%) | 1.61 (0.82 to 3.18) | 0.167 |
|  |  |  |
| Total cholesterol (mg/dL) | 1.01 (1.01 to 1.01) | 0.002 |
|  |  |  |
| Lipid-lowering agents |  |  |
| No | Ref. |  |
| Yes | 0.58 (0.43 to 0.78) | <0.001 |
|  |  |  |
| Systolic blood pressure (mm Hg) | 1.01 (1.01 to 1.02) | 0.001 |
|  |  |  |
| Antihypertensive treatment |  |  |
| No | Ref. |  |
| Yes | 0.67 (0.51 to 0.87) | 0.003 |
|  |  |  |
| Body mass index (Kg/m^2^) | 0.94 (0.90 to 0.99) | 0.009 |
|  |  |  |
| Waist circumference (cm) | 1.02 (1.00 to 1.04) | 0.050 |
|  |  |  |
| Smoking status |  |  |
| Never | Ref. |  |
| Former | 1.80 (1.31 to 2.49) | <0.001 |
| Current | 3.83 (2.61 to 5.61) | <0.001 |
|  |  |  |
| Antithrombotic treatment |  |  |
| No | Ref. |  |
| Yes | 0.94 (0.48 to 1.86) | 0.866 |
|  |  |  |
| Test of fit Hosmer-Lemeshow |  | 0.390 |
| Area under de ROC curve | 0.69 (0.67 to 0.72) | <0.001 |

**Table S4.** The multivariable logistic regression model for the presence of atheromatous disease among male subjects in the prediabetes stage.

|  | Odds Ratio  (95% Confidence Interval) | p |
| --- | --- | --- |
| Age (years) | 1.10 (1.06 to 1.13) | <0.001 |
|  |  |  |
| Glycosylated hemoglobin (%) | 2.53 (0.87 to 7.31) | 0.087 |
|  |  |  |
| Total cholesterol (mg/dL) | 1.00 (1.00 to 1.01) | 0.051 |
|  |  |  |
| Lipid-lowering agents |  |  |
| No | Ref. |  |
| Yes | 0.81 (0.52 to 1.26) | 0.348 |
|  |  |  |
| Systolic blood pressure (mm Hg) | 1.02 (1.01 to 1.03) | 0.017 |
|  |  |  |
| Antihypertensive treatment |  |  |
| No | Ref. |  |
| Yes | 1.05 (0.71 to 1.54) | 0.819 |
|  |  |  |
| Body mass index (kg/m^2^) | 0.99 (0.90 to 1.09) | 0.811 |
|  |  |  |
| Waist circumference (cm) | 1.00 (0.96 to 1.04) | 0.907 |
|  |  |  |
| Smoking status |  |  |
| Never | Ref. |  |
| Former | 2.23 (1.52 to 3.26) | <0.001 |
| Current | 9.21 (5.33 to 15.93) | <0.001 |
|  |  |  |
| Antithrombotic treatment |  |  |
| No | Ref. |  |
| Yes | 0.57 (0.19 to 1.73) | 0.323 |
|  |  |  |
| Test of fit Hosmer-Lemeshow |  | 0.602 |
| Area under de ROC curve | 0.76 (0.72 to 0.79) | <0.001 |
